# Protein-coding genes combined with long noncoding RNA as a novel transcriptome molecular staging model to predict the survival of patients with esophageal squamous cell carcinoma

**DOI:** 10.1186/s40880-018-0277-0

**Published:** 2018-04-09

**Authors:** Jin-Cheng Guo, Yang Wu, Yang Chen, Feng Pan, Zhi-Yong Wu, Jia-Sheng Zhang, Jian-Yi Wu, Xiu-E Xu, Jian-Mei Zhao, En-Min Li, Yi Zhao, Li-Yan Xu

**Affiliations:** 1 Key Laboratory of Molecular Biology in High Cancer Incidence Coastal Chaoshan Area of Guangdong Higher Education Institutes, Shantou University Medical College, Shantou, Guangdong 515041 P. R. China; 2 Department of Biochemistry and Molecular Biology, Shantou University Medical College, Shantou, Guangdong 515041 P. R. China; 3 Departments of Oncology Surgery, Shantou Central Hospital, Affiliated Shantou Hospital of Sun Yat-Sen University, Shantou, Guangdong 515041 P. R. China; 4 Institute of Oncologic Pathology, Shantou University Medical College, Shantou, Guangdong 515041 P. R. China; 5 Key Laboratory of Intelligent Information Processing, Advanced Computer Research Center, State Key Laboratory of Computer Architecture, Institute of Computing Technology, Chinese Academy of Sciences, Beijing, 100190 P. R. China

**Keywords:** Long non-coding RNA, Protein-coding gene, Esophageal squamous cell carcinoma, Overall survival, Staging model, Transcriptome

## Abstract

**Background:**

Esophageal squamous cell carcinoma (ESCC) is the predominant subtype of esophageal carcinoma in China. This study was to develop a staging model to predict outcomes of patients with ESCC.

**Methods:**

Using Cox regression analysis, principal component analysis (PCA), partitioning clustering, Kaplan–Meier analysis, receiver operating characteristic (ROC) curve analysis, and classification and regression tree (CART) analysis, we mined the Gene Expression Omnibus database to determine the expression profiles of genes in 179 patients with ESCC from GSE63624 and GSE63622 dataset.

**Results:**

Univariate cox regression analysis of the GSE63624 dataset revealed that 2404 protein-coding genes (PCGs) and 635 long non-coding RNAs (lncRNAs) were associated with the survival of patients with ESCC. PCA categorized these PCGs and lncRNAs into three principal components (PCs), which were used to cluster the patients into three groups. ROC analysis demonstrated that the predictive ability of PCG-lncRNA PCs when applied to new patients was better than that of the tumor-node-metastasis staging (area under ROC curve [AUC]: 0.69 vs. 0.65, *P *< 0.05). Accordingly, we constructed a molecular disaggregated model comprising one lncRNA and two PCGs, which we designated as the LSB staging model using CART analysis in the GSE63624 dataset. This LSB staging model classified the GSE63622 dataset of patients into three different groups, and its effectiveness was validated by analysis of another cohort of 105 patients.

**Conclusions:**

The LSB staging model has clinical significance for the prognosis prediction of patients with ESCC and may serve as a three-gene staging microarray.

## Introduction

Esophageal cancer ranks as the world’s sixth deadliest cancer and has two major histological types: adenocarcinoma and squamous cell carcinoma [1, 2]. In China, esophageal squamous cell carcinoma (ESCC) is the predominant subtype, with high incidence and poor prognosis [3]. The tumor-node-metastasis (TNM) staging system, which was proposed by the American Joint Committee on Cancer (AJCC) and the Union for International Cancer Control (UICC) in 1988 and revised in 2009, is the most important tool for determining appropriate treatment and predicting survival [4]. However, patients with ESCC at the same TNM stage may have a completely different prognosis. This is explained by the variability and heterogeneity of tumor cells [5]. Moreover, criteria used in the TNM system have varied constantly according to the different editions of the AJCC/UICC guidelines [6, 7], and the complexity of the TNM system makes it burdensome for clinicians to predict prognosis [8–10]. Therefore, a novel tumor staging or survival predicting model is urgently needed for patients with ESCC.

With the development of high-throughput sequencing technology, such as microarray analysis [11, 12], the number of gene expression profiles has rapidly increased, which provides researchers with numerous opportunities and challenges to deeply mine databanks such as the Gene Expression Omnibus (GEO) and the Cancer Genome Atlas (TCGA) thereby gaining insights into tumor staging or survival predicting models. Since Golub et al. [13] used DNA microarray technology to generate gene expression profiling data to classify acute myeloid leukemia (AML) and acute lymphocytic leukemia (ALL), numerous cancer molecular classification studies based on gene expression profiles or clinical experiments have been proposed for classifying cancer types or subtypes [14–17]. For example, The PAM50 prognostic models based on the expression of 50 genes can be applied to large series of formalin-fixed, paraffin-embedded breast cancer samples, providing more prognostic information than can be acquired from knowledge of clinical factors and immunohistochemical analysis of tumor tissues [18]. For example, analysis of the expression of the protein-coding genes (PCGs) ubiquitin-conjugating enzyme E2 C (UBE2C) and matrix gla protein (MGP) combined with two clinicopathological variables accurately predicts postoperative outcomes of patients with ESCC [19].

A new molecular staging using the G-factor, which is based on the expression of p53 and matrix metalloproteinase-7 (MMP-7), can supplement applying the TNM system to classify gastric cancer [20], and a prognostic 7-gene expression signature for stage III disease was observed in colorectal cancer [21]. Our institute has identified several prognostic molecular parameters for ESCC as well [22–29]. In particular, we have proposed a FENSAM (Fascin, Ezrin, N stage, surgery extent, activating transcription factor 3 [ATF3], M stage) model, which provides an alternative, precise classification for ESCC [16].

Similar to PCGs, certain dysregulated long non-coding RNAs (lncRNAs) act as oncogenes [30]. For example, HOX antisense intergenic RNA (*HOTAIR*) is associated with breast cancer metastasis [31]. Growth arrest specific 5 (*GAS5*) and LINC00538 (*Yiya*) are promising prognostic biomarkers for liver metastases in patients with early-stage colorectal cancer [32]. An 8-lncRNA expression signature was identified in esophageal cancer, which may provide more significant prognostic information beyond conventional clinicopathological factors [33].

Here we used two microarray datasets and relevant clinical information from the GEO dataset to explore the association between PCGs and lncRNAs and the survival of patients with ESCC. For this purpose, we constructed a PCG-lncRNA transcriptome staging model to predict the prognosis of patients with ESCC.

## Materials and methods

### GEO data

PCG and lncRNA expression data and clinical data of corresponding patients with ESCC were obtained from the publicly available GEO database (https://www.ncbi.nlm.nih.gov/geo/). We excluded cases without clinical survival information. Two ESCC microarray datasets (GSE53624 and GSE53622) generated using the Agilent-038314 CBC *Homo sapiens* lncRNA + mRNA microarray V2.0 (http://www.genomics.agilent.com/) were selected. We divide GSE53624 into the training and test dataset randomly. The GSE53624 test dataset and GSE53622 was used for internal validation. The main clinicopathological characteristics of patients are summarized in Table 1.

**Table 1:** Clinicopathological characteristics of patients with esophageal squamous cell carcinoma

Variable	The GEO datasets^a^			The experimental set		
	Number of patients	5-year OS rate (%)	*P* value^b^	Number of patients	5-year OS rate (%)	*P* value^b^
Total	119			105		
Age (years)						
≤ 59	89	48.31	0.03	63	58.73	0.69
> 59	90	33.33		42	54.76	
Sex						
Female	33	33.33	0.30	25	48.00	0.16
Male	146	42.47		80	60.00	
Tumor location						
Upper thorax	20	25.00	0.10	7	71.4	0.15
Middle thorax	97	40.21		54	61.11	
Lower thorax	62	46.78		44	50.00	
Histological grade						
G1	49	28.57	0.02	15	60.00	0.16
G2	98	44.90		73	64.29	
G3	32	46.88		17	47.06	
Primary tumor						
T1	12	41.67	0.04	21	71.42	0.03
T2	27	40.74		63	63.49	
T3	110	44.55		21	31.58	
T4	30	26.67				
Regional lymph nodes						
N0	83	56.62	< 0.01	50	68.00	< 0.01
N1	62	27.41		32	56.25	
N2	22	27.27		16	37.50	
N3	12	25.00		7	28.50	
pTNM stage						
I	10	70.00	0.00	6	33.30	0.00
II	77	53.25		48	70.80	
III	92	27.17		51	47.05	
Adjuvant therapy						
Unknown	45	64.44		0		
No	30	30.00	0.50	43	58.14	0.70
Yes	104	33.65		62	56.45	
Radiotherapy	Unknown			18	48.83	0.11
Chemotherapy	Unknown			19	59.09	
Radiotherapy + chemotherapy	Unknown			25	48.00	

*OS* overall survival

^a^Comprising the GSE52634 and GSE53622 datasets

^b^Log-rank test was used

### Probe re-annotation pipeline

The GPL18109 probe set sequences for the Agilent-038314 CBC *Homo sapiens* lncRNA + mRNA microarray V2.0 were downloaded from the Agilent website (https://www.agilent.com/). PCG and lncRNA expression data from the Agilent-based expression profile of ESCC cohorts (GSE53624 and GSE53622) was obtained by re-annotating microarray probes according to the sequences of the probe sets and the annotations of PCG and lncRNA records in GENCODE (GRCh38, release 21, http://www.gencodegenes.org/). We used BLASTn (ftp://ftp.ncbi.nlm.nih.gov/blast/executables/LATEST/) to align the probe sequences to those of noncoding and coding transcript sequences from GENCODE.

The alignments were filtered as follows: (i) only probes perfectly matched to a transcript were retained, resulting in two sets of probes targeting protein-coding and -non-coding transcripts, respectively; (ii) probes targeting noncoding transcripts that perfectly matched cDNA coding sequences were removed; (iii) all transcripts corresponding to the retained probes were mapped to the genome and annotated as PGCs or lncRNAs.

### Sample collection and preparation

Besides above 179 ESCC patients from GEO database, we collected 105 ESCC patients as the experimental set from the Chaoshan District of Guangdong Province, which has a high prevalence of ESCC [26]. The experimental set was used for external validation. Samples were collected from the Department of Oncological Surgery of the Central Hospital of Shantou City, P.R. China between February 2012 and December 2013. Tumor and paired nontumor tissues were collected from patients who underwent surgical resection. After examination by a pathologist, tissues were immediately frozen in liquid nitrogen and stored at − 80 °C. Partial tissue samples were used for hematoxylin and eosin staining to confirm the diagnosis and analysis of pathological grade, metastasis, and tumor cell content. Tumor samples contained > 80% tumor tissue free of necrosis were selected. Only those died of ESCC were included in the study. The follow-up for patients after esophageal resection continued until death, and it extended to March 2016. We excluded patients suffering from severe postoperative complications, other tumors, or those who died of other causes. The clinical data were available in Table 1. Cases were classified according to the TNM classification of the International Union Against Cancer, 7th edition. Evaluation of tumor differentiation was based on the guidelines of the World Health Organization (WHO) Pathological Classification of Tumors. Overall survival (OS) was defined as the interval between surgery and death from tumors or the last observation of surviving patients. The study was approved by the Ethics Committees of the Central Hospital of Shantou City and Shantou University Medical College. Written informed consent to use resected samples for research purposes was obtained from all patients.

### Reverse transcription (RT) and real-time PCR

Total RNA was extracted using TRIzol (15596-018, Life Technologies, Grand Island, NY, USA) and purified using a PureLink RNA Mini Kit (12183018A; Life Technologies) according to the manufacturer’s protocol. The purity and concentration of RNA were determined according to the ratio of absorbance of 260 nm/280 nm light using a NanoDrop ND-2000 spectrophotometer (ND-2000, Thermo Fisher Scientific, Waltham, MA, USA). The cDNA synthesis was performed by reverse transcription using random hexamer primers (Takara, Dalian, Liaoning, China). Real-time PCR was performed using a SYBR Premix Ex Taq kit (DRR037A, DRR081A; Takara). Briefly, reverse transcription was performed at 37 °C for 15 min and at 85 °C for 5 s. Real-time PCR was performed using an ABI 7500 real-time PCR system (Life Technologies) as follows: 95 °C, 30 s; 95 °C, 5 s; 60 °C, 34 s (30 cycles). Relative quantification of mRNA expression was calculated using the 2^−ΔΔCt^ method. Quantitative RT-PCR (qRT-PCR) was performed in triplicate and repeated at least three times, as described previously [34, 35]. All methods were performed in accordance with guidelines and regulations of the ethics committees identified above.

### Statistical analysis

All analyses were performed using the R program (www.r-project.org), including packages named pROC, rpart, and survival downloaded from Bio-conductor (http://www.bioconductor.org/). Univariate Cox regression analyses were used to identify common PCGs and lncRNAs that associated with OS. *P *< 0.05 indicates a statistically significant difference. All insignificant PCGs and lncRNAs with *P *>0.05 were excluded. We performed principal component analysis (PCA) [36–38] to reduce expression data and to capture innate patient characteristics.

We used the R NbClust package (Euclidean distance, complete linkage), and it provides 30 indices for determining the number of clusters. The output of the software proposes the best clustering scheme from the results obtained by varying combinations of the clusters, distance measures, and clustering methods. These operations identify the optimal number of clusters by calculating several cluster indices. The clusters were validated using the Calinski and Harabasz index [39, 40].

Kaplan–Meier survival analysis was performed to test the equality of survival distributions of different groups for each ESCC cohort, and statistical significance was assessed using the two-sided log-rank test. Further, time-dependent receiver operating characteristic (ROC) curves were used to compare the sensitivity and specificity of the survival prediction, and the area under the curve (AUC) value was calculated from the ROC curve. Multiclass ROC curves were used to evaluate the effectiveness of multi classification predictions, which were calculated using the R packages called pROC [41, 42]. Classification and regression tree (CART) analysis was performed using a Recursive Partitioning and Regression Trees (RPART) library in R to develop a risk-staging model and to determine predictors of survival from the set of survival-related PCGs and lncRNAs [43, 44]. This is a nonparametric statistical method that uses a series of dichotomous splits to create a decision tree.

**Fig. 1:**
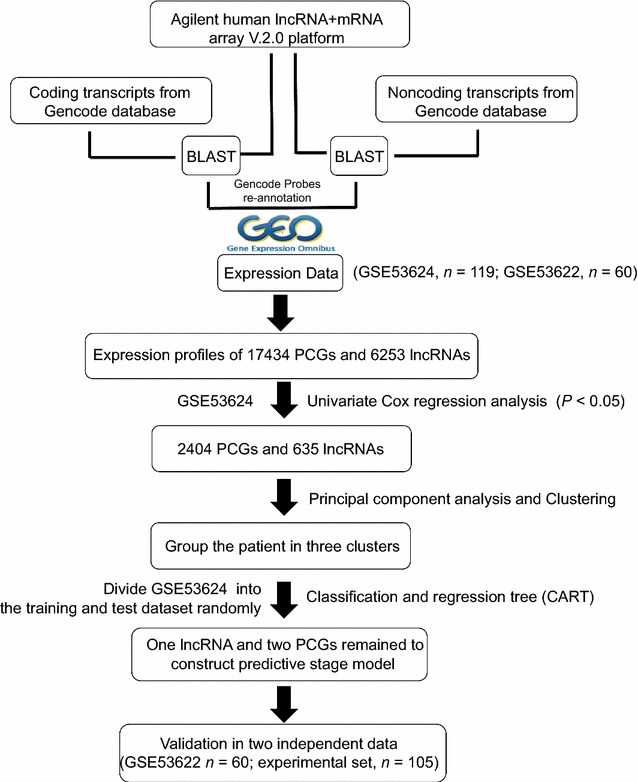
Schedule of the analyses used to develop the transcriptome molecular staging model and validate its predictive efficiency. *PCG* protein-coding gene, *lncRNA* long non-coding RNA

To begin the CART analysis, patients identified from the GSE53624 dataset were randomly split into the training and testing groups using the function “sample” of the R program [45]. CART was applied first to the training group and then to the test sample to assess the model’s generalizability and to evaluate the overfitting of the model. When the classification tree was generated, error tests and pruning were performed to construct the final tree of parameters with the best size, lowest misclassification rate, and lowest complexity. The selection process of the prognostic model is shown in Fig. 1.

The associations between coexpressed lncRNA and PGCs in the molecular staging model were computed using Pearson correlation coefficients visualized with Cytoscape. We next performed Gene Ontology (GO) and Kyoto Encyclopedia of Genes and Genomes (KEGG) enrichment analyses of the coexpressed protein-coding genes to predict biological functions using the ClueGo plugin of Cytoscape (version 3.2.3) [46], which is a commonly used functional annotation tool that can assess over-representation of functional categories among a gene set of interest. Enrichment analysis, which was performed using the functional annotation chart and functional annotation clustering options, was limited to GO terms and KEGG pathways in the “Biological Process” categories. Functional annotations with *P* < 0.05 were considered significant [47].

## Results

### Selection of ESCC microarray datasets and acquisition of PCG and lncRNA expression values

According to the dataset screening criteria described in Methods, 179 samples (119 from the GSE53624 dataset and 60 from the GSE53622 dataset) were selected. All these ESCC samples contained tumor tissues and adjacent normal tissues. Probe reannotation of the Agilent-038314 CBC *Homo sapiens* lncRNA + mRNA microarray V2.0 identified 17,434 PCGs and 6253 lncRNAs. We retained probes that were uniquely mapped to the genomic coordinates of PCGs or lncRNAs derived from GENCODE. Multiple probes (or probe sets) mapping to the same PCGs or lncRNAs were integrated using the arithmetic mean of the values of multiple probes (or probe sets) and were used to generate new PCG and lncRNA expression profile values from the GSE53624 and GSE53622 datasets. Further, we used fold-change values (cancer/normal) on a log2 scale in the next analysis.

### Selection of prognostic PCGs and lncRNAs from the GSE53624 dataset

**Fig. 2:**
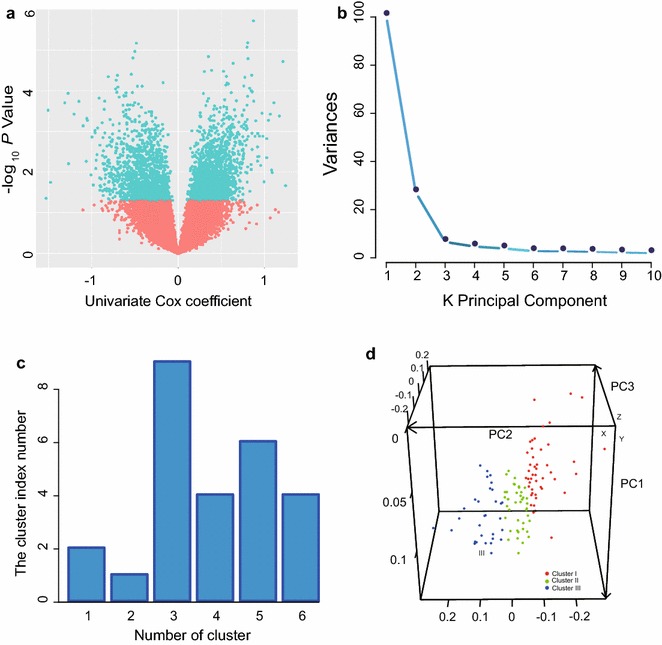
The patients identified from the GSE53624 dataset (*n *= 119) are grouped with three risk stages. **a** Univariate Cox proportional hazards regression analysis of the expression profiling data of PCGs and lncRNAs. **b** Eigenvalues of the principal components show most of the variance in the GSE53624 dataset is contained in the first three principal components. **c** Clustering of the patients with ESCC identified from the GSE53624 dataset according to the three principal component scores using NbClust (Euclidean distance, complete linkage) indicates that optimal cluster number was three with the largest index. **d** Principal component analysis of the GSE53624 dataset. Axes are principal components 1, 2, and 3. *PCG* protein-coding gene, *lncRNA* long non-coding RNA

The ESCC patient cohort from GSE53624 (*n* = 119) was selected to explore the association of OS with PCGs or lncRNAs. We conducted univariate Cox proportional hazards regression analysis of the PCG and lncRNA expression profiling data, with OS as the dependent variable, and identified 2404 PCGs and 635 lncRNAs that significantly associated with OS (*P *< 0.05) (Fig. 2a), which were therefore considered potential prognostic markers.

### Identification of three distinct clusters of patients with ESCC

To identify genes that are more important for staging and to reduce the dimensionality of the profile with2404 PCGs and 635 lncRNAs, PCA was performed. This analysis demonstrated that the survival-related PCG-lncRNA set in GSE63624 was reduced to three independent principal components PC1, PC2, and PC3, accounting for 99% of the variance of the component space (Fig. 2b). Using the three PC scores as variables, we applied the NbClust clustering procedure to cluster the patients with ESCC identified from the GSE53624 dataset and highlighted a three-cluster partition as the best, with the largest cluster index number (Fig. 2c, d).

### Association of patient groups with survival

**Fig. 3:**
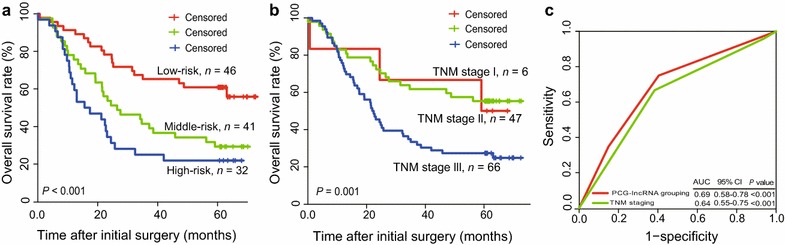
Survival prediction power of PCG-lncRNA grouping versus TNM staging for patients identified from the GSE53624 dataset. **a** Kaplan–Meier analysis of patient survival when the PCG-lncRNA grouping is applied. **b** Kaplan–Meier analysis of patient survival when TNM staging is applied. **c** Comparison of the PCG-lncRNA grouping and the TNM staging systems using ROC analysis. *PCG* protein-coding gene, *lncRNA* long non-coding RNA

Kaplan–Meier analysis revealed that the prognostic PCGs and lncRNAs had the potential to classify the 119 patients into three groups with different OS estimates. Patients in the high-risk group had shorter OS compared with those in the middle- and low-risk groups (median OS: 16.5 months vs. 26.5 months and 50.9 months, *P *< 0.05) (Fig. 3a). The 5-year OS rate was approximately 20% in the high-risk group, approximately 30% in the middle-risk group, and > 50% in the low-risk group.

### Comparison of the PCG-lncRNA grouping versus TNM staging in survival prediction

According to Kaplan–Meier analysis, the PCG-lncRNA grouping yielded a better classification of patients compared with that of the TNM staging system (Fig. 3b). To compare the sensitivity and specificity in survival prediction between TNM staging and PCG-lncRNA grouping, we performed ROC analysis. In the GSE53624 dataset, the predictive ability of the PCG-lncRNA grouping was better than that of TNM staging (AUC: 0.69 vs. 0.65, *P* < 0.05) (Fig. 3c).

### Construction of the LINC01800-SEMA3A-BEX2 (LSB) staging model

Kaplan–Meier and ROC analyses showed that the PCG-lncRNA grouping improved the classification of patients, indicating its value as a novel, efficient staging plan. We next pursued identifying markers to classify the three groups as follows. We randomly divided the GSE53624 dataset into a training set (*n* = 59) and a testing set (*n* = 60) for internal validation. Next, we selected the first 100 genes with the highest absolute loading, e.g., the 100 genes with the highest positive or negative correlation corresponding to each principal component. The clinical attributes of patients, such as age, sex, tobacco use, alcohol use, tumor location, tumor grade, T stage, N stage, and TNM stage, were used as variables to perform the CART routines.

**Fig. 4:**
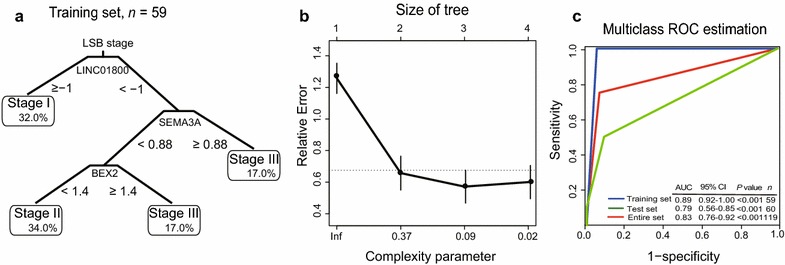
The LSB staging model comprising SEMA3A, BEX2, and LINC01800 selected using classification and regression tree (CART) analysis. **a** SEMA3A, BEX2, and LINC01800 form the classification tree generating using CART analysis. The percentage represents the proportion of patients at every LSB stage in the training set. **b** Test error result of the classification tree. **c** Multiclass ROC analysis was performed in the training set, test set, and entire GSE53624 dataset

Subsequently, CART analysis of the training set generated the final tree composed by LINC01800, semaphorin 3A (SEMA3A), and brain-expressed X-linked 2 (BEX2) identified from the survival-related PCGs and lncRNAs. Moreover, there were no clinical attributes of patients with lowest error rate remaining in the classification tree produced using RPART (Fig. 4a, b; Table 2). Higher expression levels of BEX2 and LINC01800 were associated with longer OS (univariable Cox regression coefficient < 0). Higher expression levels of SEMA3A were associated with shorter OS (univariable Cox regression coefficient > 0). Multiclass ROC was used to evaluate the predictive ability of the LSB (stands for the first letter of each of the three genes) staging model, and the AUC was 0.89 (*P* < 0.05) in the training set.

**Table 2:** Identified PCGs and lncRNAs and their associations with prognosis

Ensemble ID	Gene symbol	Gene name	Chromosome location	Coefficient^a^	*P* value^a^	Gene expression level association with prognosis
ENSG00000075213	SEMA3A	Semaphorin 3A	Chromosome 7: 83955777–84492724 (−)	0.17	0.01	High
ENSG00000133134	BEX2	Brain-expressed X-linked protein 2	Chromosome X: 103309346–103311046 (−)	− 0.22	0.01	Low
ENSG00000234572	LINC01800		Chromosome 2: 64846130–64863626 (−)	− 0.20	0.00	Low

*PCG* Protein-coding gene, *lncRNA* long non-coding RNA

^a^Derived from univariable Cox regression analysis of the GSE53624 dataset

The LSB staging model was used to stratify patients in the test set (*n *= 60) and entire GSE53624 dataset (*n *= 119), and the AUC values were 0.79 and 0.83 (*P* < 0.05), indicating that this model efficiently stratified patients into different prognostic groups (Fig. 4c).

**Fig. 5:**
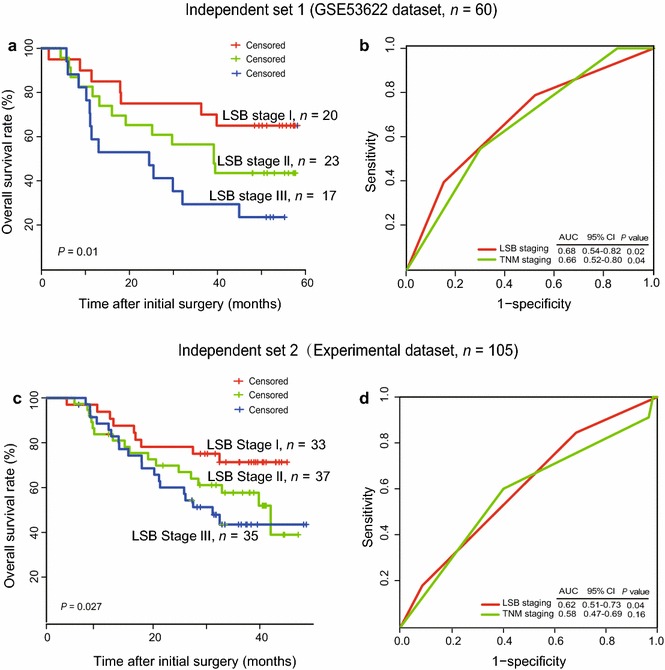
Validation of the LSB staging model using the GSE53622 dataset (*n* = 60) (**a**, **b**) and experimental dataset (*n* = 105) (**c**, **d**). Kaplan–Meier analysis and comparison of the LSB staging model and the TNM staging using ROC analysis

To validate its power for staging efficiency, the LSB staging model was evaluated using an independent dataset (GSE53622, *n *= 60). Kaplan–Meier survival curves for patients with LSB I, II, and III ESCC, which were classified according to the respective cutoff values of the three molecular markers, are shown in Fig. 5a (median OS: 56.7 vs. 39.2 vs. 24.5 months, *P* = 0.01). The 5-year OS rates were of was 63.4% in patients with LSB I ESCC, 39.6% in patients with LSB II ESCC, and 21.2% in patients with LSB III ESCC. The percentages of patients with LSB I, LSB II, and LSB III ESCC were 33.3%, 38.3% and 28.4%, respectively, similar to those in the training group (LSBs I-III: 32.0%, 34.0%, and 34.0%, respectively). Moreover, the AUC of the LSB staging model was 0.68, greater than 0.66 for the TNM staging (*P* < 0.05) (Fig. 5b).

### Validation of the LSB staging model using an experimental dataset

To confirm the findings described above, RNA was extracted from 105 pairs of tissues from patients with ESCC, reversely transcribed, and quantified using real-time RT-PCR. The primer sequences for SEMA3A, BEX2, and LINC01800 cDNAs for real-time RT-PCR are shown in Table 3. Beta-actin mRNA was used as the internal control. Integrating the qRT-PCR results and clinical data with the LSB staging model, the 105 patients were classified into LSB I, II, and III groups, with different OS (median OS: 31.1 months vs. 42.0 months vs. 53.4 months, *P *< 0.05) (Fig. 5c). The predictive ability of the LSB staging method was better than that of the TNM staging (AUC: 0.62 vs. 0.58) (Fig. 5d).

**Table 3:** Primer sequences used for real-time RT-PCR

Gene	Forward (5′–3′)	Reverse (5′–3′)
SEMA3A	TGGTTCTGCATGTTCTCGCT	CTCTCTGCGACTTCGGACTG
BEX2	TCGAGAATCGGGAGGAGGAGAC	TCCTGGTTGACATTTTCCACGAT
LINC01800	CCACACTGGAGTGCAGCTAT	CCACCTGTCTGATGGTCTTCT
Β-actin	AGCGAGCATCCCCCAAAGTT	GGGCACGAAGGCTCATCATT

*RT-PCR* Reverse transcription polymerase chain reaction

### Functional classifications of the LSB genes

**Fig. 6:**
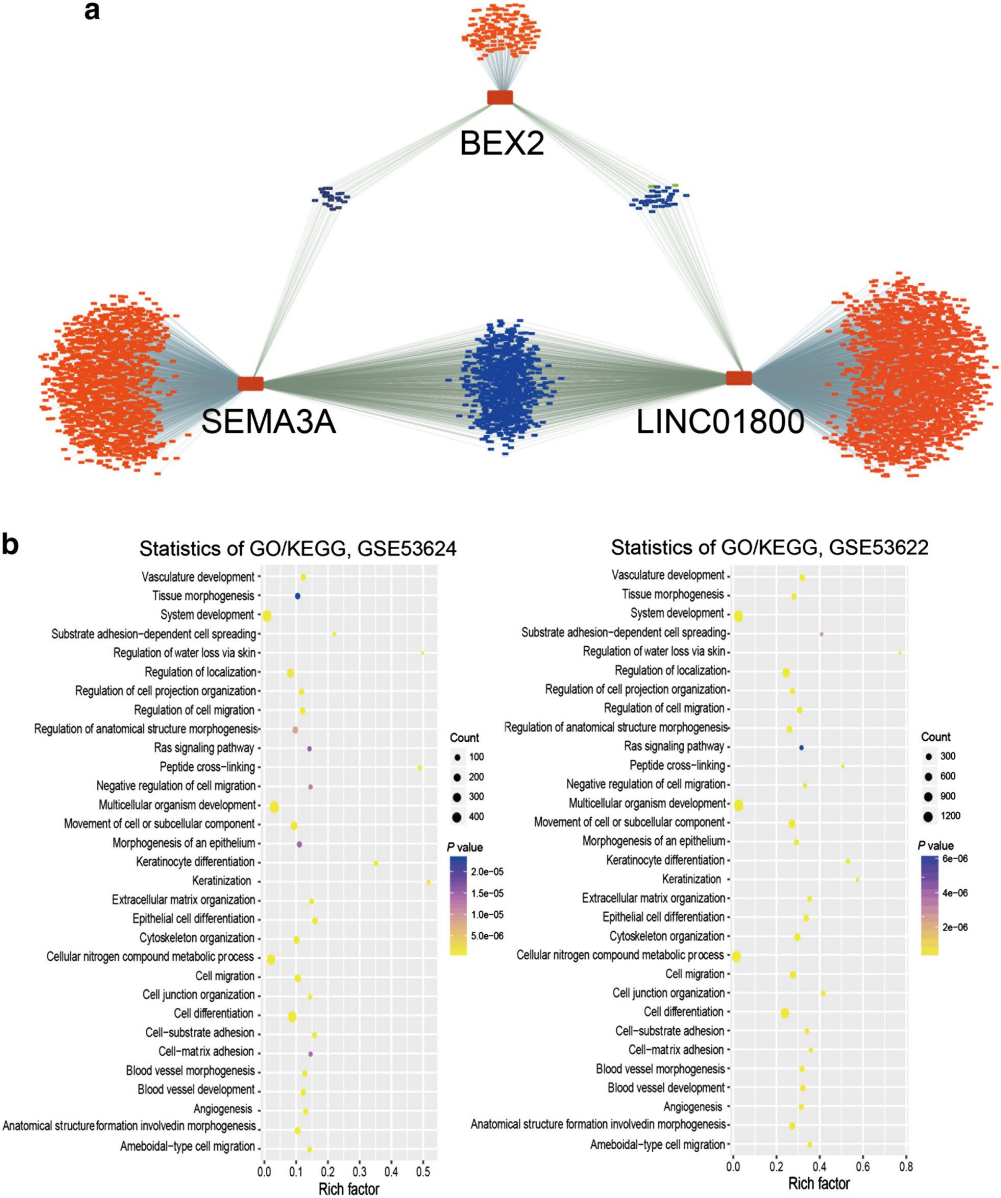
Coexpression network analysis and prediction of the function of SEMA3A, BEX2, and LINC01800. **a** Coexpression network of SEMA3A, BEX2, and LINC01800 with other genes in the GSE53624 and GSE53622 datasets (Pearson correlation coefficient > 0.5, *P *< 0.05). Blue or red genes were coexpressed with two or one of the three identified genes in the LSB staging model, respectively. **b** Functional enrichment of the protein-coding genes which were coexpressed with SEMA3A, BEX2, and LINC01800, using ClueGo

To further investigate the potential biological roles of the three markers, a coexpression network comprising SEMA3A, BEX2, and LINC01800 was constructed by computing Pearson correlation coefficients of the GSE53624 and GSE53622 datasets (Fig. 6a). GO and KEGG analysis of the PCGs which were coexpressed with LINC01800, SEMA3A, and BEX2 revealed that in the two datasets, the coexpressed PCGs were significantly enriched in 28 GO terms and 3 KEGG pathways (*P *< 0.05). These findings suggest that SEMA3A, BEX2, and LINC01800 may be involved in tumorigenesis through interacting with those coexpressed PCGs that influence biological processes such as angiogenesis, cell migration, cell differentiation, and cell adhesion (Fig. 6b).

## Discussion

Using advances in microarray technology, algorithms, and data accumulated for ESCC, we constructed the LSB staging model comprising SEMA3A, BEX2, and LINC01800. We employed an unsupervised learning algorithm called PCA and CART based on reannotating the Agilent-038314 CBC *Homo sapiens* lncRNA + mRNA microarray V2.0 [47, 48]. The LSB staging model was simpler to use with higher prediction accuracy compared with the ESCC staging model comprising ubiquitin-conjugating enzyme E2 C (UBE2C) and matrix gla protein (MGP) gene expression levels [19] and TNM staging or the FENSAM (Fascin, Ezrin, N stage, surgery extent, activating transcription factor 3 [ATF3], M stage)staging model constructed in our previous study [16]. Briefly, the LSB staging model performed better than the TNM staging system and other staging models of ESCC, according to our data re-mining. With the rapid increase of related studies, more transcriptome staging models will become available, such as combining PCGs and lncRNAs with microRNAs or circular RNAs or both.

CART analysis is a powerful statistical method with significant clinical utility. The tree-building technique can be used to construct predictive models by testing the influence of variables on the “outcome.” Compared with standard methods such as multivariate regression, CART analysis is highly advantageous, because it analyzes highly skewed data, with simplicity and clarity. Therefore, we performed CART analysis for model development to generate a classification tree. In this tree, the complexity parameter reflects the tradeoff between tree complexity and how well the tree fits the data. After error testing and pruning, the final tree comprising LINC01800, SEMA3A, and BEX2 achieved the best size, lowest misclassification rate, and smallest complexity parameter. We validated the effectiveness of the LSB staging model using the GSE53622 dataset and a cohort of 105 patients’ tissues from our laboratory.

Interestingly, any two nodes in the LSB staging model had conjunct coexpressing genes, but no intersection of the three. LINC01800, SEMA3A, and BEX2 and their coexpressed genes contribute to angiogenesis, cell migration, cell differentiation, and cell adhesion, which was revealed through analysis of GSE53624 and GSE53622 datasets. BEX2 is overexpressed in a subset of primary breast cancers and mediates the inhibition of apoptosis of breast cancer cell lines through nerve growth factor/nuclear factor kappa-light-chain-enhancer of activated B cells (NF-κB) [49]. Overexpression of SEMA3A promotes tumor progression and predicts poor prognosis of patients with hepatocellular carcinoma after curative resection [50], consistent with its similar effect on OS of patients with ESCC in the present study. However, SEMA3A expression decreases significantly as gastric cancer progresses and metastasizes, suggesting that SEMA3A may serve as a candidate tumor suppressor [51].

Certain limitations to this study, other than the limited robustness of the data for ESCC need to be acknowledged. First, only a fraction of human lncRNAs (6253 of > 15,000) and PCGs (17,434 of > 30,000) were included in the analysis. Therefore, the prognostic lncRNA and PCGs identified here may not represent all candidates. Second, experimental studies on these three genes may provide important information that will enhance our understanding of their functional roles. Third, the expression values of microarrays differ from those of RT-PCR [52, 53]. Therefore, we used corresponding LSB stage ratios of the training dataset and the ranks of the experimental 2^−ΔΔCt^ values to group patients, in contrast to the same cutoff values of the three genes in microarray. Thus, the data were insufficient to confirm cutoff values of the three genes in RT-PCR. Nevertheless, the robustness of our LSB staging model using independent and experimental datasets indicates that this model has potential clinical significance for translation to the clinic as a three-gene microarray, likely to serve as a powerful prognostic staging model for ESCC.

In conclusion, we show here that the LSB staging model can accurately predict the survival of patients with ESCC. Moreover, the method used to construct the LSB staging model suggests a general strategy and effective methodology that will facilitate research aimed at identifying new clinical staging markers for other types of cancer.
